# Role of NFAT5 in the Immune System and Pathogenesis of Autoimmune Diseases

**DOI:** 10.3389/fimmu.2019.00270

**Published:** 2019-02-19

**Authors:** Naeun Lee, Donghyun Kim, Wan-Uk Kim

**Affiliations:** ^1^Center for Integrative Rheumatoid Transcriptomics and Dynamics, The Catholic University of Korea, Seoul, South Korea; ^2^Department of Microbiology and Immunology, Seoul National University College of Medicine, Seoul, South Korea; ^3^Department of Biomedical Sciences, Seoul National University College of Medicine, Seoul, South Korea; ^4^Institute of Infectious Diseases, Seoul National University College of Medicine, Seoul, South Korea; ^5^Division of Rheumatology, Department of Internal Medicine, The Catholic University of Korea, Seoul, South Korea

**Keywords:** NFAT5, hyperosmolarity, immune regulation, autoimmune diseases, therapeutic target

## Abstract

The nuclear factor of activated T cells (NFAT5), also known as a tonicity-responsive enhancer-binding protein, was originally identified as a key transcription factor involved in maintaining cellular homeostasis against hypertonic and hyperosmotic environments. Although NFAT5 has been expressed and studied in various types of hyperosmolar tissues, evidence has emerged that NFAT5 plays a role in the development and activation of immune cells, especially T cells and macrophages. The immune-regulatory function of NFAT5 is achieved by inducing different target genes and different signaling pathways in both tonicity-dependent and -independent manners. Particularly in response to hyperosmotic stress, NFAT5 induces the generation of pathogenic T_H_17 cells and pro-inflammatory macrophages, contributing to autoimmune and inflammatory diseases. Meanwhile, with tonicity-independent stimuli, including activation of the Toll-like receptors and inflammatory cytokines, NFAT5 also can be activated and promotes immune cell survival, proliferation, migration, and angiogenesis. Moreover, under isotonic conditions, NFAT5 has been implicated in the pathogenesis of a variety of inflammatory and autoimmune diseases including rheumatoid arthritis. This review describes the current knowledge of NFAT5, focusing on its immune-regulatory functions, and it highlights the importance of NFAT5 as a novel therapeutic target for chronic inflammatory diseases.

## Introduction

The nuclear factor of activated T cells-5 (NFAT5), also known as tonicity-responsive enhancer binding protein (TonEBP), was initially identified from the kidney medulla as a central regulator of cellular response to ambient hypertonicity (1). Under physiological conditions, the operation of urinary concentrating mechanisms in the kidney medulla often causes high osmolality—about four times that of the blood or higher—thereby maintaining constant body fluid volume and blood pressure (2). However, excess extracellular osmolality to renal cells drives double-strand DNA breaks and cell death (3). Fortunately, the high osmotic stress increases the amount and the nuclear translocation of NFAT5 at once that is directly responsible for accumulating organic osmolytes to restore homeostasis in the kidney medulla (1). Transcription factor NFAT5 induces the expression of a variety of osmoprotective genes, including ion transporters, aldose reductase, and heat shock protein 70, for intracellular distribution of compatible osmolytes and urea (4). The reduced intracellular ionic strength and urea concentration contribute to protecting the cells within the kidney medulla from the deadly effects of hypertonicity (4).

## Gene, Protein, and Molecular Characteristics of NFAT5

The human *Nfat5* gene exists in chromosome 16q22.1, and the mouse homolog is found in chromosome 8D (5). Various transcripts are made from the gene by alternative promoters and alternative splicing; thus far, 16 different variants have been reported. Among these, 12 transcripts have protein-coding potential, and the remaining variants appear not to encode proteins (6). The NFAT5 transcript is expressed in various human tissues such as the kidney, brain, heart, thymus, lung, and skeletal muscle (1, 7). In contrast to the ubiquitous mRNA expression, the abundant expression of NFAT5 protein is detected only in extract from the thymus, while there are much lower amounts in the testes, lung, liver, and brain and no expression in other tissues including lymph nodes (8).

**Figure 1:**
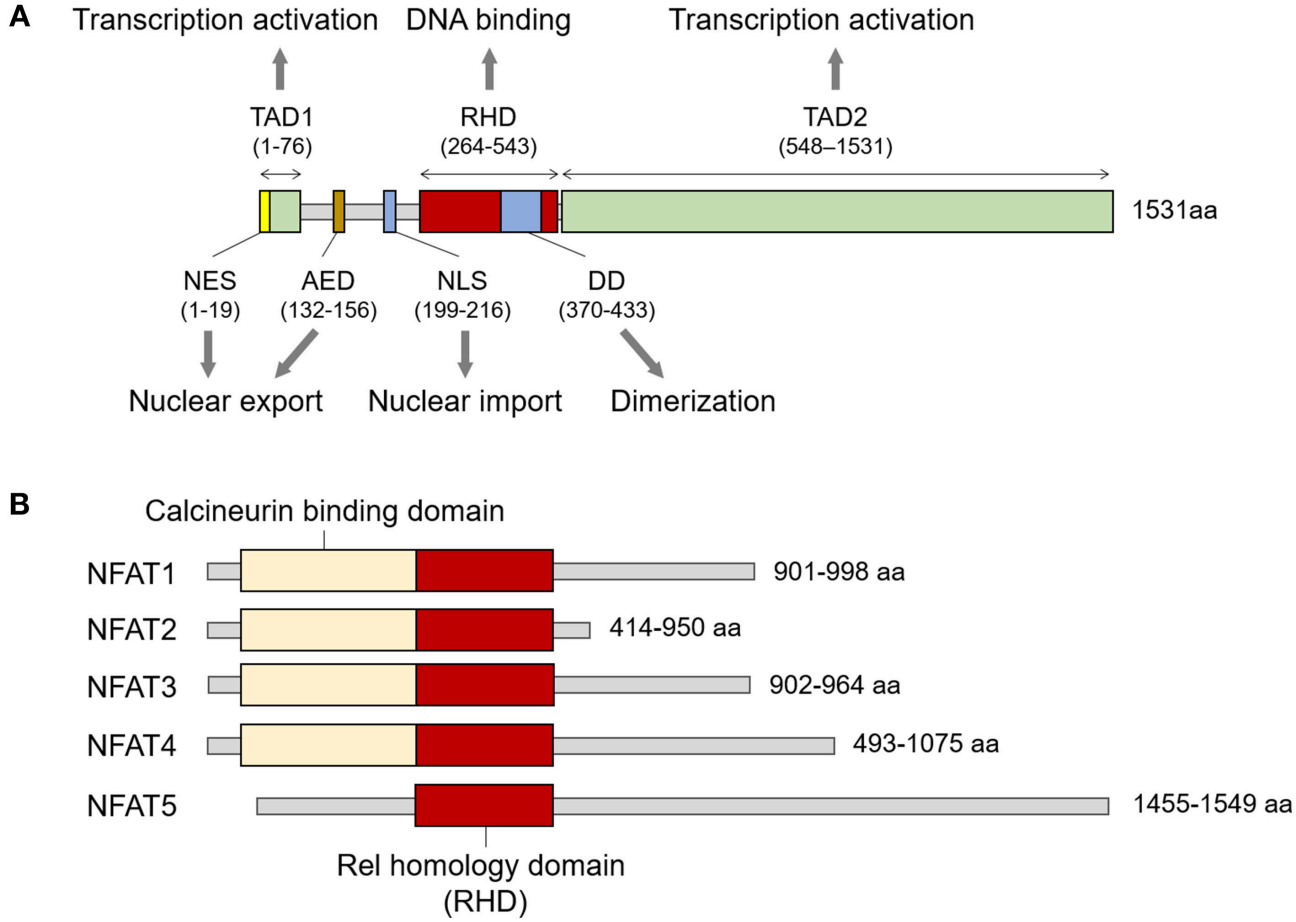
Structure of NFAT family members. **(A)** Signature domains in human NFAT5 protein and their function. NFAT5 contains several functional domains including NES, TAD1, AED, NLS, RHD, DD, and TAD2. The number of amino acids in parenthesis is based on KIAA0827 (1531 amino acid; GenBank accession no. AB020634). **(B)** Comparison of representative structures between NFAT members. All NFAT members share the Rel homology domain, but only NFAT5 does not have a calcineurin-binding domain. The minimum and maximum numbers of amino acids among representative transcript variants are presented.

NFAT5 belongs to the Rel family, in which members share the Rel-homology domain (RHD) responsible for DNA binding (Figure 1A) (9). The RHD of NFAT5 is highly similar to those of other NFAT members (NFAT1 to 4) but has minimal amino acid identity with that of NFκB (9). In addition to the RHD, the protein structure consists of a leucine-rich canonical nuclear export sequence (NES) located at the first 19 amino acids, an N-terminal compositionally serine/threonine and proline-rich region (transactivation domain 1; TAD1), an auxiliary export domain (AED), a consensus bipartite nuclear localization signal (NLS), a dimerization domain (DD) within the RHD, and a C-terminal low-complexity region (glutamine and serine/threonine-rich region, a TAD2) (10–13) (Figure 1A). However, NFAT5 protein lacks docking sites for calcineurin that is necessary for the nuclear translocation of the other NFAT proteins (Figure 1B), and thus the calcium/calcineurin signaling cascade is dispensable for activating the transcription factor.

NFAT5 protein exists in both cytoplasm and nucleus under isotonic conditions, but it is not in a static state; rather, it is in an active equilibrium state between cytoplasmic and nucleic protein (1, 11). The active nucleocytoplasmic shuttling under isotonic conditions is mediated by NLS and NES that are recognized by specific import and export receptors, respectively (11). Changes in extracellular tonicity rapidly alter the amount and ratio of NFAT5 between the cytosol and nucleus. Hypertonicity induces the transcription and translation of NFAT5 as well as leads to the translocation and accumulation of NFAT5 into nucleus (1, 4). As in isotonic condition, the NLS is indispensable for the nuclear import process induced by hyperosmotic stress (10). In contrast, hypotonic condition predominantly leads to the nuclear export of the transcription factor, which depends on the presence of AED but not of NES (11).

NFAT5 recognizes and binds to TGGAAANNYNY (N, any nucleotide; Y, any pyrimidine) sequences on DNA promoters similar to those recognized by NFAT1 (7, 9). NFAT5 acts as a dimeric form, as does NF-κB, and does not cooperate with FOS or JUN, whereas other NFAT members exist as a preformed monomer and often bind DNA together with Fos or Jun (9, 14).

## Role of NFAT5 in the Immune System

### Tonicity-Dependent Immune Regulation

#### High Salt and Th17 Polarity

The intake of too much dietary salt (sodium chloride, NaCl) has been implicated in various diseases including hypertension, stroke, coronary heart disease, heart failure, and renal disease (15, 16). In numerous instances, the disorders related to high salt intake are accompanied by the induction of T_H_17 cells that have a pathogenic role in the progression of chronic inflammatory and autoimmune diseases (17–19). When T_H_17 polarization was induced *in vitro* from human naïve CD4^+^ T cells by cytokines, a modest increase in NaCl concentration drove further augmentation in IL-17A and GM-CSF secretion (18). Even in healthy adults, dietary salt consumption gradually increases T_H_17 cells and reciprocally decreases Treg cells, although the T_H_17/Treg ratio is somewhat low on the first day of high salt loading (20).

**Figure 2:**
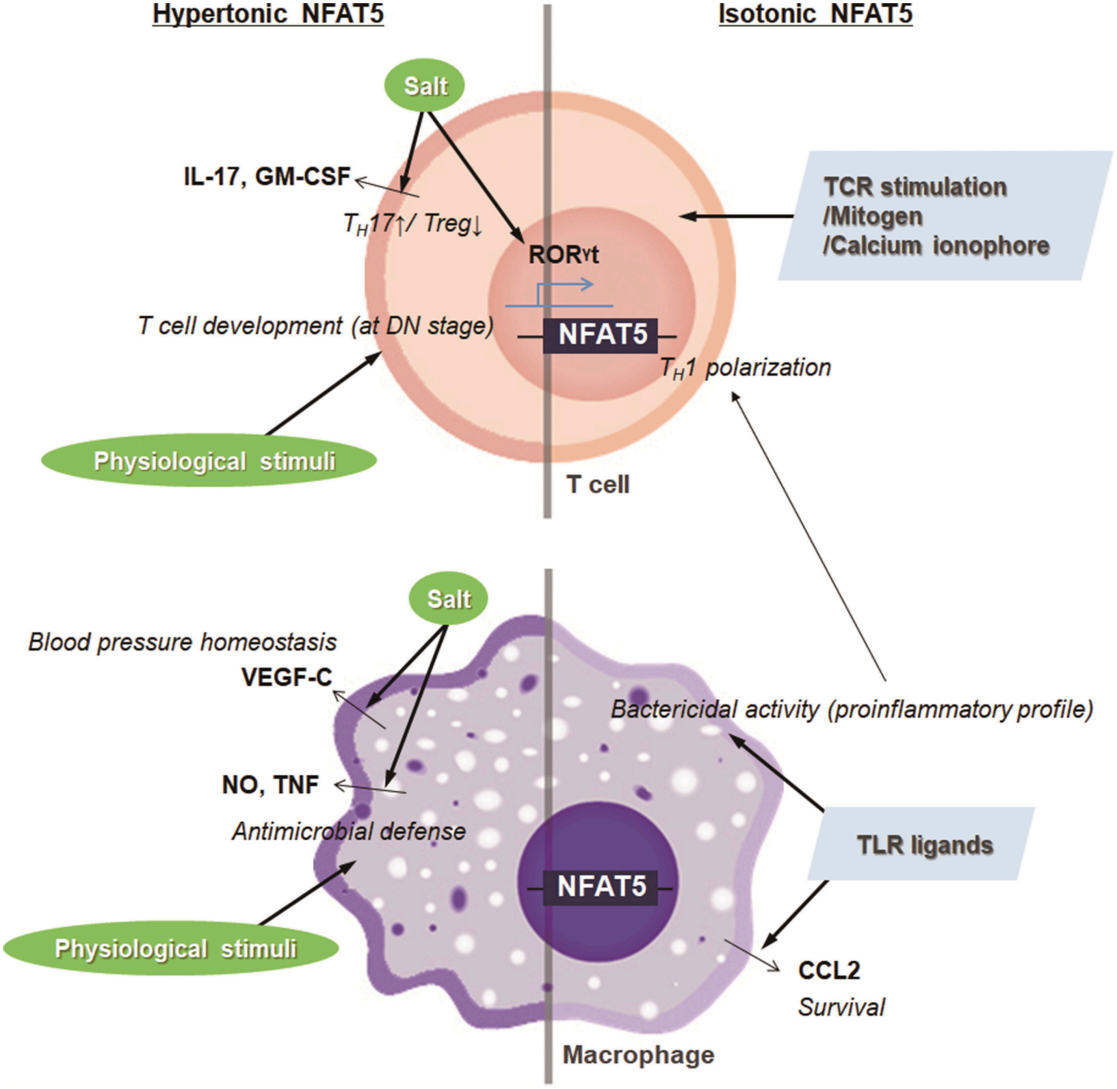
Targets and immunologic functions of hypertonic and isotonic NFAT5. NFAT5 induced by hypertonic or isotonic stimuli maintains blood pressure homeostasis, induces T cell development and T_H_17 cell differentiation, and enhances antimicrobial activity and cell survival of macrophages. In addition, NFAT5-mediated proinflammatory alteration in macrophages skews CD4^+^ T cells to T_H_1 polarization.

Salt is a typical dietary nutrient that increases tonicity (15). Thus, simultaneously with the increase in T_H_17 cell population, the high salt consumption leads to the expression and transactivation of NFAT5. Moreover, several groups have shown that NFAT5 activation is pivotal in high salt-mediated T_H_17 polarization (18, 21). Gene silencing of *Nfat5* in CD4^+^ T cells abrogates the expression of T_H_17-associated genes including *Il17* and *Ror*γ*t* (18, 21). Altogether, the high salt-NFAT5-T_H_17 axis may explain the pathogenesis of several inflammatory and autoimmune diseases (Figure 2).

#### High Salt and Innate Immunity

The hyperosmolar state induced by salt intake also stimulates the expression and translocation of NFAT5 in innate cells, affecting physiologic and pathologic immune responses. In the macrophages, NaCl leads to the NFAT5-mediated induction of nitric oxide and TNF, which promotes cutaneous antimicrobial defense (Figure 2) (22). Also, under conditions of high salt intake, osmotic stress-induced NFAT5 contributes to the expression of vascular endothelial growth factor (VEGF)-C in macrophages (23). The VEGF-C restores blood pressure homeostasis by increasing the density and hyperplasia of the lymph capillary network, which protects against hypertension (Figure 2) (23). In invariant nature killer T cells, high salt condition inhibits TCR-dependent and TCR-independent IFN-γ production, which is dependent on NFAT5 (24). Although earlier researchers emphasize the protective and physiologic roles of NFAT5 in maintaining immune defense and body homeostasis, NFAT5 sometimes can critically mediate pathologic immune responses and tissue damage as well. For example, high extracellular NaCl concentration induces NLRP3 gene expression in retinal pigment epithelial cells that is partially dependent on the activities of NFAT5 (25); the retinal inflammation is thought to be associated with the development of age-related macular degeneration (25).

#### Physiological and Pathophysiological Osmotic Stress and Immune Regulation

Osmolality within lymphoid microenvironment, like the thymus and spleen, is significantly higher than that of serum, indicating that lymphocytes are exposed to physiologic osmotic stress (26). Moreover, sera obtained from NFAT5-deficient mice are markedly hypertonic and contain abnormally high concentrations of sodium, suggesting that NFAT5 might function as part of an osmotic stress response (27, 28). Simultaneously, NFAT5-haplo-deficient mice exhibited a 30–50% reduction in thymic and splenic cellularity evenly distributed among T cell subsets (26, 28). A similar phenotype was observed in transgenic mice which express a dominant inhibitory form of NFAT5 only in T lymphocytes by using the CD2 promoter (29). The decrease in thymic cellularity is associated with the reduced number of peripheral T cells (29). Consistently, conditional deletion of NFAT5 at the early double-negative thymocyte stage causes smaller thymi, fewer double positive and single positive thymocytes, and decreased cellularity in the lymphoid organ, although mice deficient in NFAT5 after double-positive thymocyte stage (*CD4-Cre*;*Nfat5*^fl/fl^ mice) exhibit normal plasma tonicity and have only a mild naïve/memory imbalance in T cells (28, 30). Taken together, NFAT5 induced by physiological osmotic stress serves a survival signal during T cell development (Figure 2). As another example, renal sodium gradient induced by the physiological and pathophysiological situation causes chemokine secretion by the renal tubular epithelium, which recruits circulating monocyte-derived mononuclear phagocytes into the hypertonic region and stimulates antimicrobial activity of renal macrophages (31). Interestingly, NFAT5 is involved not only in the migration activity toward hypertonic spot but also in the antimicrobial function of renal macrophages (Figure 2) (31, 32). These findings indicate that NFAT5 has a pivotal role in regulating immune system in response to physiological and pathologic osmotic stress.

### Tonicity-Independent Immune Regulation

In addition to the enhanced extracellular tonicities, isotonic factors other than tonicity are involved in NFAT5 abundance and activity in some cells and tissues that are presumably well-conserved by isotonic body fluids (27). Isotonic stimuli-induced NFAT5 has been implicated in various physiological and pathological responses that have no relevance to osmotic stress. For example, several researchers have confirmed that NFAT5 plays a pivotal role in the pathogenesis of RA, a tonicity-independent disorder, using various experimental arthritis models (33, 34). Upon toll-like receptor (TLR) stimulation in the macrophages, NFAT5 is activated through xanthine oxidase-reactive oxygen species (ROS)-p38MAPK cascade (33). The protein provides apoptotic resistance to RA macrophages by inducing CCL2 secretion (Figure 2) (33). Like that in macrophages, inflammatory stimuli-induced NFAT5 activation promotes the expression of tissue factor and CCL2 in the FLSs of RA patients (RA-FLSs), causing the hypermotility and invasiveness that are pathological features of RA (35). Next, during vascular injury, platelets, and other cells of the vasculature secrete platelet-derived growth factor-BB (PDGF-BB), which drives the proliferation and migration of vascular smooth muscle cells (SMCs) (36). PDGF-BB increases NFAT5 protein expression and its reporter activity in SMCs, which mediates SMC migration (36). Angiotensin II, another factor involved in vascular injury, promotes SMC contraction and hypertrophy (36). The contractile SMC phenotype is dependent on the angiotensin II-mediated NFAT5 nuclear import and its transcriptional activity (36). Some inflammatory molecules, such as TNF-α, have been known to cause intervertebral disc degeneration, triggering chronic back pain (37). In the process, the inflammatory responses to TNF-α in NP cells are mediated by induced NFAT5 transcription (37). Two growth factors, bone morphogenetic protein 2 (BMP-2) and transforming growth factor-β (TGF-β), were found to significantly increase both NFAT5 protein expression and transactivation in nucleus pulposus cells located at the center of the intervertebral disc, which influences disk cell function through chondroitin sulfate and aggrecan synthesis (38). Inflammatory stimuli such as lipopolysaccharide (LPS), IFN-γ, and IL-4 also induce the expression and activation of the transcription factor in microglia, resident immune cells of the central nervous system (39). In the same context, ischemic brain injury induces NFAT5 expression, which is required for achieving an osmotic equilibrium (39, 40). Besides, clustering of α6β4 integrin in carcinomas enhances NFAT5 protein and hence NFAT5-dependent transcription, which promotes carcinoma invasion into surrounding tissues (41). NFAT5 is also required for systemic inflammation and septic shock associated with NF-κB enhanceosome in myeloid cells (42). LPS stimulation after classical or alternative polarization in macrophages enhances NFAT5 expression (43). The increased transcription factor in macrophages enhances the proinflammatory profile such as bactericidal capability and promotes more dominant Th1 polarization compared with Th2 responses (Figure 2) (43). In T lymphocytes, isotonic stimuli, such as cross-linking with T cell receptors, mitogen, and calcium ionophore, are also able to induce NFAT5 expression (Figure 2) (8). Taken together, regardless of osmotic stress, various physiological and pathophysiological stimuli trigger the NFAT5 expression and activity implicated in various immune responses and disease pathogenesis (Figure 2).

### Context-Dependent Regulation and Functions of NFAT5

Interestingly, NFAT5 induced by isotonic stimuli (isotonic NFAT5) and osmotic NFAT5 shows distinct differences in the lymphocytes. For instance, in hypertonic culture media, the proliferation of NFAT5-deficient CD4^+^ T cells was markedly reduced as compared to that of normal CD4^+^ T cells, resulting from osmoadaptive functions of NFAT5 (44). In contrast, there were no differences in T cell proliferation regardless of NFAT5 expression under isotonic culture condition (44). In addition, NFAT5 induced by high salt media skews CD4^+^ T cells toward the T_H_17 phenotype (18, 21), whereas under isotonic normal media, the depletion of NFAT5 in CD4^+^ T cells enhances interferon γ (IFNγ) and IL-17 expression and reduces Treg responses (21). Like T cells, NFAT5 has a critical role in the B-cell proliferation induced by LPS treatment under hypertonic conditions but not isotonic media (45). In macrophages, both LPS and NaCl reciprocally inhibit the expression of downstream target genes (46). In this process, NFAT5 contributes to context-dependent inhibition through the activation of different ROS sources (46).

It is unclear how exactly isotonic NFAT5 differs from osmotic NFAT5 with regard to its functional and molecular outcomes. As mentioned above, we suggested that ROS function as molecular sensors to discriminate between isotonic and osmotic stimuli, directing NFAT5 activity toward differential responses in a context-dependent manner (46). Alternatively, cell proliferation-inducing stimuli, including PDGF, TGF-β, and TNF-α, may increase in active macromolecular biosynthesis, leading to a hyperosmolar state, albeit very transiently, in the cells stimulated with such stimuli. Although hypertonic stress may not approach that within the kidney, another possibility is that environmental osmotic stress might be exacerbated by the depletion of intracellular osmolytes and reduction in available cell volume caused by the massive induction of intracellular protein biosynthesis during cellular hyperproliferation. An earlier finding that the liver, a non-proliferative but metabolically active tissue, shows hyperosmolarity supports this notion (27). Despite these possibilities, the exact difference between isotonic and osmotic NFAT5 in the regulation and function still remains to be unveiled.

## Role of NFAT5 in the Pathogenesis of Autoimmune and Inflammatory Diseases

### NFAT5 and Chronic Arthritis

RA is a chronic inflammatory disease characterized by the expansion of inflammatory immune cells as well as synoviocytes, leading to the destruction of bone and cartilage (47, 48). RA can present with numerous autoimmune features, including producing autoantibodies such as rheumatoid factors and anti-citrullinated protein antibodies (ACPA) (47, 48). Although the etiology of RA remains elusive, interplay between genetic susceptibility and environmental factors seems to trigger disease onset. Environmental risk factors such as smoking, air pollutants, and the microbiome can drive the epigenetic modification, increasing the risk for developing RA in individuals with genetic susceptibility, including certain MHC (e.g., HLA-DR1 and DR4) or non-MHC molecules associated with autoimmune responses (47). In particular, cigarette smoking, one of the most prominent environmental risk factors for RA, increases citrullination to self-proteins and the formation of ACPA (48). As a result of autoimmune responses to citrullinated proteins, a variety of immune cells including macrophages, B cells, and T cells heavily infiltrate the synovial tissues and are involved in the initiation and perpetuation of RA. These cells can activate each other, constructing a very complex inter- and intracellular network of pro-inflammatory cytokines and chemokines including IL-1β, TNF-α, IL-6, and CCL2 (47). Chronic exposure to these pro-inflammatory cytokines, growth factors, and hypoxia may lead to abnormal proliferation of synovial fibroblasts, a pathologic hallmark of RA, and confer on these cells apoptotic resistance (35).

Recent researchers have demonstrated that NFAT5 activation by tonicity-independent, isotonic stimuli is a key regulator of RA pathology. Masuda et al. first identified that NFAT5 mRNA is expressed in the RA synovia, particularly at sites of bone destruction (49). Along with this, our group has shown that NFAT5 is highly expressed in the RA-FLSs and that its expression in RA-FLSs is upregulated by the stimulation of pro-inflammatory cytokines such as IL-1β, TNF-α, and TGF-β (33, 35). Through the global gene expression profiling of RA-FLSs, we also found that NFAT5 is a major transcription factor for regulating the migration and invasion, an aggressive phenotype of RA-FLSs, which are mediated by the upregulation of CCL2- and tissue factor expressions as its downstream target genes (35). Thus, we presume that NFAT5 plays an essential role in promoting RA-FLS migration and invasion.

Transcriptomic analysis of RA macrophages revealed that NFAT5 is also a master regulator for the pathology of RA macrophages (34). In that analysis, differentially expressed genes (DEGs) in RA macrophages overlapped significantly with the DEGs governed by NFAT5 (34). The overlapping DEGs represented a variety of biologic processes such as cell proliferation, apoptosis, and death (34). Moreover, NFAT5-deficient macrophages showed reduced survival and proliferation, suggesting that NFAT5 promotes the survival of macrophages (34). Additionally, as mentioned earlier, NFAT5 in macrophages can be activated by isotonic TLR ligands (e.g., LPS) as well as high salt (33, 50). TLR-stimulated NFAT5 activates a sets of downstream target genes including *Nos2, Il6*, and *Tnf* that differ from osmolarity-associated target molecules, leading to macrophage activation and TLR-induced arthritis (33). Along with this, NFAT5-deficiency reduces disease severity in mice with chronic inflammatory arthritis dependent on TLR-2 and 4 (51). Taken together, we consider that NFAT5 is an important regulator of macrophage activation and survival and that it thereby contributes to RA pathology.

Of interest, recent studies have shown that smoking together with high sodium intake is associated with the development of ACPA-positive RA (52, 53). As described in the discussion of High salt and T_H_17 polarity, sodium chloride drives the development of pathogenic T_H_17 cells through the serum glucocorticoid kinase 1 (SGK1)-mediated pathway; SGK1 is an inducible salt-sensing kinase (18, 54). Conversely, high salt prevents the suppressive function of Foxp3^+^ Treg cells (55). These reports provide important evidence that high salt itself favors the polarization of immune cells, including T helper cells, toward pro-inflammatory phenotypes while suppressing the activation of Treg, promoting immune-mediated diseases. Although the role of NFAT5 in high salt-induced development of T_H_17 cells in autoimmune diseases remains elusive, it is conceivable that tonicity-dependent NFAT5 activation has a pivotal role in initiating RA in smokers, who often enjoy eating salty food because it is crucial to both high salt-induced activation of T_H_17 cells (56) and osmo-protection (57).

Taken together, given the effects of NFAT5 on immunity under isotonic and hypertonic conditions, we believe that the pathologic role of NFAT5 in RA is not limited to a single type of immune cell. Rather, overall RA pathology can be demonstrated to result from the combinatory action of NFAT5 on multiple types of cells including macrophages, T_H_17 cells, Treg cells, and synoviocytes.

### NFAT5 and Diabetes Mellitus

Type 1 diabetes (T1D) is well-known as a T-cell mediated autoimmune disorder characterized by destroying insulin-producing β cells in the pancreas, which causes multiple complications such as retinopathy and nephropathy (58). T1D as a metabolic disorder interrupts blood glucose homeostasis, resulting in hyperglycemia owing to a lack of insulin. Hyperglycemia induces metabolic influx associated with increased osmotic stress, which leads to the development of diabetes-related microvascular complications (59). As discussed above, hyperosmotic stress itself functions as a potent inflammatory stimulus to release pro-inflammatory cytokines (60). In terms of tonicity-dependent mechanisms, intracellular osmolarity tightly regulates the expression of aldose reductase (AR), which is a key inducer of hyperglycemia-induced oxidative stress as well as a target gene of NFAT5 under hypertonic conditions in both peripheral blood mononuclear cells and human mesangial cells (59). Consistently, in streptozotocin-induced diabetic retinopathy, NFAT5 deficiency decreases AR expression and alleviates the retinopathy (61). Furthermore, NFAT5 deficiency attenuates insulin resistance and reduces macrophage infiltration to adipose tissues in mice with high-fat diet- or streptozotocin-induced diabetes (62).

In terms of tonicity-independent mechanisms, the progression of T1D accompanies impaired immune tolerance and subsequent aberrant immune activation. Several studies in animal models showed that T1D could be induced by the interaction among several types of immune cells, including CD4^+^, CD8^+^ T cells, and macrophages. In particular, CD8^+^ T cells are mainly involved in destroying pancreatic β cells through diverse pathways, including IFN-γ production and expression of death receptor FAS (58). IFN-γ produced by CD8^+^ T cells can drive IL-1β and TNF-α production in macrophages, leading to β cell apoptosis (58). In contrast, Treg cells counterbalance the activation of effector T cells to prevent the onset of T1D (63, 64). For example, studies in a Treg cell-deficient animal model showed broken immune tolerance and exacerbated disease development under diabetes-prone conditions (65). Thus, enhancing the function of Treg cells may be suggested as a potential approach to treating T1D (65). Recently, a new mechanism by which microRNA181a activity reduced the induction of Treg cells to break peripheral tolerance was revealed in a T1D animal model (66). Of interest, miRNA181a promotes the expression of NFAT5 in a tonicity-independent manner, which substantially reduced the frequency of Treg cells. The authors proposed that microRNA181a-mediated NFAT5 interferes with inducing Treg cells and thereby contributes to T1D (66).

Collectively, through tonicity-dependent and -independent mechanisms, NFAT5 regulates the development of T1D, another model of autoimmune disease, which seems to be mediated by the induction of AR (under hypertonic conditions) and expansion of Treg cells (under isotonic conditions). In this respect, targeting NFAT5 signaling using siRNA or small molecules may limit islet autoimmunity.

### NFAT5 and Multiple Sclerosis

Multiple sclerosis (MS) is an autoimmune disease of the central nervous system (CNS) that is also triggered by several environmental factors in genetically susceptible individuals. Similar to RA, autoreactive T cells play a central role in MS pathogenesis; in particular, pathogenic T_H_17 cells have been implicated in the disease progression (67). Recent studies have suggested that high salt induces the generation of IL-17-producing CD4^+^ T_H_17 cells in the CNS and exacerbates the development of experimental autoimmune encephalomyelitis (EAE), a mouse model of MS dependent on T_H_17 cells (18, 54). Similar to the pathogenic pathway of RA, high salt results in increased p38-MAPK and NFAT5 expression in this model, promoting the expression of SGK1, a downstream target of NFAT5 (54, 57), indicating that EAE is dependent on a high salt-NFAT5-SGK1- T_H_17 axis. In parallel with the findings in animal models, sodium concentrations in urine from MS patients shows a positive correlation with disease progression, although there was no association between sodium intake and onset risk in pediatric MS patients (57, 68). Taken together, although further investigation is required, aforementioned studies highlight the importance of osmotic NFAT5 in the pathogenesis of MS.

### NFAT5 and Tumor Immunity

In addition to autoimmune diseases, accumulating evidence demonstrates that NFAT5 is associated with the progression of various types of tumors, including breast cancer, hepatocellular carcinoma, and lung cancer (69–71). As mentioned above, it has been revealed that high salt promotes inflammatory responses by inducing a variety of pro-inflammatory mediators, including inducible nitric oxide synthases, IL-6, and TNF-α in innate immune cells. Additionally, high salt directly induces the expression of VEGF in breast tumor cells, resulting in angiogenesis and immune dysfunction (70). Interestingly, sodium concentrations in breast tumors were shown to be higher than those in normal cells (70, 72). Accordingly, NFAT5, a representative osmo-sensitive transcription factor, has been proposed to mediate tumor progression. Indeed, a positive correlation was found between the expression of NFAT5 and inflammatory breast cancer (73). NFAT5 expression is dramatically elevated in hepatocellular carcinoma and lung adenocarcinoma cells compared with normal tissues (69, 71). Moreover, inhibiting NFAT5 expression reduces the proliferation and migration of lung adenocarcinoma cells, which is accompanied by suppressing the expression of aquaporin-5 (AQP5), a water transporter depending on the osmotic gradient (69). It also attenuates hepatocellular carcinogenesis, recurrence, and metastasis (71). It is implicated in the relationship of NFAT5 expression in tumor cells and tumorigenesis in terms of tonicity-dependent manners.

López-Rodríguez et al. recently published a tonicity-independent mechanism in which NFAT5-sufficient macrophages drive a pro-inflammatory function to promote IFNγ-expressing T_H_1 polarization via up-regulating IL-12 production (43). In contrast, NFAT5-deficient macrophages exhibit reduced pro-inflammatory function, resulting in enhanced tumor growth in Lewis lung carcinoma and ID8 ovarian carcinoma models (43). Intriguingly, NFAT5-deficient macrophages show the reduced infiltration of effector CD8^+^ T cells into the tumor site (43), which is accordant with our findings of NFAT5 regulation of FLS migration (35). Together, in a sharp contrast to the tumorigenic role of NFAT5 in the cancer cells, NFAT5 in immune cells seems to escalate antitumor immunity by polarizing pro-inflammatory macrophages as well as promoting accumulation of CD8^+^ T cells adjacent to tumor masses. Therefore, in light of the dynamic interaction of cancer cells with microenvironments, it should be judiciously determined what the net *in vivo* effect of NFAT5 inhibition for cancer progression is.

## Perspectives on NFAT5 as a Therapeutic Target of Autoimmune and Inflammatory Diseases

Here, we review the essential role of NFAT5 in immunity and autoimmune diseases. Given the pivotal role of NFAT5 in macrophage activation and survival, Treg cell generation, and T_H_17 differentiation, it is an appropriate therapeutic target for autoimmune diseases. Indeed, we have demonstrated that transfection of NFAT5 siRNAs into RA synoviocytes and endothelial cells inhibits their proliferation and survival and impedes angiogenic processes (35, 51). Again, mice injected with NFAT5 siRNAs showed reduced streptozotocin-induced diabetic retinopathy (61), suggesting that transcriptional knockdown of NFAT5 using siRNAs can be a useful strategy to treat NFAT5-dependent autoimmune inflammatory diseases including RA and diabetes mellitus.

Some drugs or drug candidates also have been investigated to test their capacity to inhibit NFAT5 expression and/or activation. For example, metformin, a well-known anti-diabetic drug, inhibits the expression of hypertonicity-induced NFAT5 and its downstream target genes, and it enhances the apoptosis of renal medullary interstitial cells in T2D mice (74). Recently, our research group identified two small molecules, KRN2 and KRN5, to specifically suppress NFAT5 expression via a high-throughput screening system; KRN2 is 13-(2-fluorobenzyl)-berberine, a derivative of berberine, and KRN5 is an oral derivative of KRN2 (50). KRN2 selectively inhibits the transcriptional activation of NFAT5 by blocking NF-κB binding to the NFAT5 promoter region, downregulating the expression of pro-inflammatory NFAT5-target genes in macrophages including *Nos2* and *Il6* (50). Importantly, KRN2 selectively inhibited the activation of inflammatory NFAT5 induced by TLR ligands without hampering high-salt-induced NFAT5 and its target gene expressions (50). Intra-peritoneal or oral administration of KRN2 and KRN5 markedly suppresses the progression of chronic inflammatory arthritis (50). Moreover, KRN2 effectively inhibits the pro-migratory capacity of synoviocytes induced by TGF-β as well as suppressing NFAT5 expression in FLSs (35), suggesting that KRN2 can be a potential therapeutic agent to block FLS invasiveness. Subsequently, Serr at al. demonstrated that injecting KRN2 into diabetes-prone mice dramatically promoted peripheral Treg cells and alleviated the infiltration of auto-immune inflammatory cells in the pancreas (66). Interestingly, KRN2 decreases NFAT5 expression in an infiltrated T cell-specific manner and has no adverse effects on metabolic parameters.

**Figure 3:**
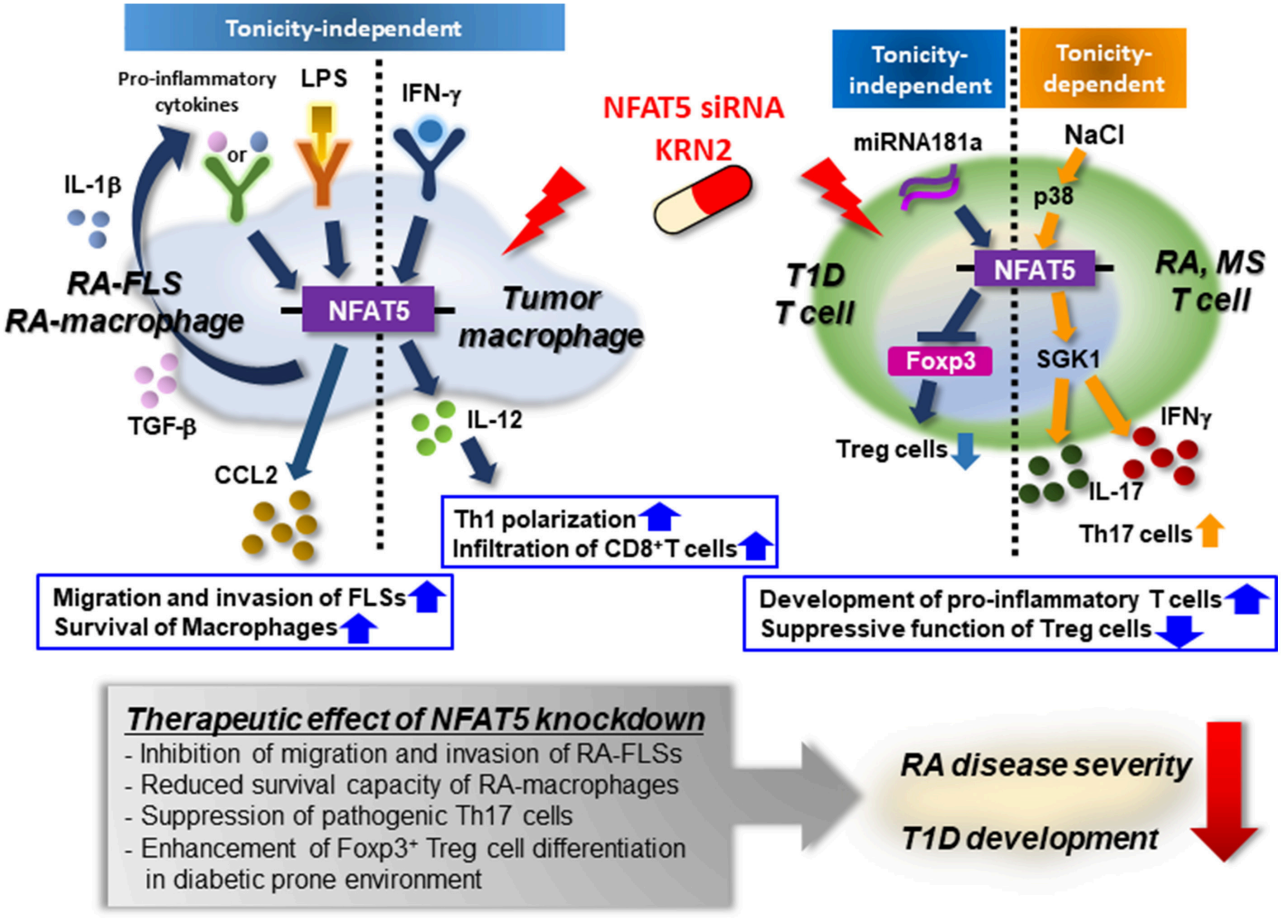
Mode of action of NFAT5 as a therapeutic target for autoimmune diseases. Pro-inflammatory cytokines (e.g., IL-1β and TGF-β) or TLR ligand (e.g., LPS) enhances the expression of NFAT5 in rheumatoid arthritis (RA)-FLSs and RA macrophages. Up-regulated NFAT5, in turn, produces CCL2, promoting RA-FLS migration and invasion and conferring on RA-macrophages apoptotic resistance. IFN-γ promotes NFAT5-dependent IL-12 production, which mediates T_H_1 polarization, and the infiltration of CD8^+^ T cells into tumor microenvironments. High salt also increases the expression of NFAT5 via the p38-mediated pathway, resulting in the development of pro-inflammatory T cells that produce IL-17 and/or IFNγ. Furthermore, in diabetes-prone mice, NFAT5 suppresses the differentiation of Foxp3^+^ Treg cells, breaking peripheral tolerance. As a therapeutic target, NFAT5 siRNA or KRN2 prevents the pathologic action of NFAT5 and alleviates the progression of RA and T1D. Therefore, NFAT5 blockades may be considered a promising strategy to treat autoimmune diseases, including RA and T1D.

Taken together, we anticipate that anti-NFAT5 blockades (e.g., small molecules including KRN2 or KRN5 and small hairpin RNAs) will be novel candidates in the treatment of autoimmune diseases such as RA, T1D, and MS in which NFAT5 and its targets play a key role. The immunological roles of NFAT5 and the action of NFAT5 siRNA or KRN2 under pathological conditions is summarized in Figure 3.

## Author Contributions

NL, DK, and W-UK designed the concept of the review. NL and DK wrote the manuscript and depicted the figures. W-UK provided critical revisions to the final manuscript. All authors approved the final version of the manuscript.

### Conflict of Interest Statement

The authors declare that the research was conducted in the absence of any commercial or financial relationships that could be construed as a potential conflict of interest.
